# The *Non-Coding RNA* Journal Club: Highlights on Recent Papers

**DOI:** 10.3390/ncrna1010087

**Published:** 2015-06-17

**Authors:** Manuela Ferracin, Daniel Gautheret, Florent Hubé, Sendurai A. Mani, John S. Mattick, Ulf Andersson Ørom, Gaetano Santulli, R. Keith Slotkin, Zofia Szweykowska-Kulinska, Joseph H. Taube, Franck Vazquez, Jian-Hua Yang

**Affiliations:** 1 Department of Morphology, Pathology Oncology and Experimental Biology Section, University of Ferrara, Ferrara 44121, Italy; E-Mail: manuela.ferracin@unife.it; 2 Laboratory for Technologies of Advanced Therapies, University of Ferrara, 44121 Ferrara, Italy; 3 Institute for Integrative Biology of the Cell (I2BC), CEA, CNRS, Université Paris-Sud, 91198 Gif sur Yvette, France; E-Mail: daniel.gautheret@u-psud.fr; 4 CNRS UMR7216, Epigenetics and Cell Fate, Université Paris Diderot, Sorbonne Paris Cité, Bâtiment Lamarck B, Case Courrier 7042, 35 rue Hélène Brion, 75013 Paris, France; E-Mail: florent.hube@univ-paris-diderot.fr; 5 Department of Molecular Pathology, The University of Texas MD Anderson Cancer Center, 77054 Houston, TX, USA; E-Mails: smani@mdanderson.org (S.A.M.); jhtaube@mdanderson.org (J.T.); 6 Garvan Institute of Medical Research, Sydney, New South Wales, Australia, and St Vincent’s Clinical School, University of New South Wales, Sydney, 2010 New South Wales, Australia; E-Mail: j.mattick@garvan.org.au; 7 Noncoding RNA Research Group, Max Planck Institute for Molecular Genetics, 14195 Berlin, Germany; E-Mail: oerom@molgen.mpg.de; 8 Department of Physiology and Cellular Biophysics, Clyde and Helen Wu Center for Molecular Cardiology, College of Physicians and Surgeons, Columbia University Medical Center, New York, 10032 NY, USA; E-Mail: gs2620@cumc.columbia.edu; 9 Department of Molecular Genetics, The Ohio State University, Columbus, 43210 OH, USA; E-Mail: slotkin.2@osu.edu; 10 Department of Gene Expression, Faculty of Biology, Institute of Molecular Biology and Biotechnology, Adam Mickiewicz University, Umultowska 89, 61-614 Poznan, Poland; E-Mail: zofia.szweykowska-kulinska@amu.edu.pl; 11 MDPI AG, *Non-Coding RNA* Editorial Office; Klybeckstrasse 64, 4057 Basel, Switzerland; 12 Key Laboratory of Gene Engineering of the Ministry of Education, State Key Laboratory for Biocontrol, Sun Yat-sen University, 510275 Guangzhou, China; E-Mail: yangjh7@mail.sysu.edu.cn

## 1. Introduction

The number of papers dealing with new *modus operandi* or new biological functions of non-coding RNAs published in recent years has indeed exploded. A simple search for ‘non-coding RNA’ in Pubmed on 10 June 2015 yielded 128,649 articles, half of which were published in the last 10 years [1]. Every researcher in this field knows that he has something to learn and can discover new ideas, new concepts or new tools from studies made in models others than the ones used in its lab.

The Scientific board of *Non-Coding RNA* publishes here its first *Journal Club* and highlights, in about hundred words, a selection of the most interesting papers published recently. We hope we will tease your curiosity and encourage you to read full papers outside of your research area that you may not have read otherwise.

The Lauressergues *et al.* paper [2] attracted a lot of attention and raised new important questions, which you will see from different perspectives in Section 9.

The *Non-Coding RNA* Scientific Board wishes you a good reading!

## 2. EMT-Regulated Circular RNAs

Highlight by Joseph H. Taube and Sendurai A. Mani

While circular RNAs (circRNAs)—sequential exons spliced to each other in a circular arrangement—have been known since the 1990s, regulators of their expression have remained elusive. Using a model of epithelial-mesenchymal transition (EMT) in mammary epithelial cells, Conn *et al*. show that circRNAs are not universally-produced (detected from only 22% of transcripts) and that following EMT, circRNAs are more abundant and half of them are undetected prior to EMT [3]. Screening for regulators using siRNA-knockdown showed that the RNA-binding protein, QKI, interacts with sequence-specific elements to promote circRNA formation. Since QKI is upregulated by EMT, this protein and its target circRNAs are prime candidates for functional roles in tumor progression.

## 3. Myoregulin Is a Novel Modulator of Calcium Handling and Excitation–Contraction Coupling: Hunting for Easter Eggs in the Concealed Microproteome

Highlight by Gaetano Santulli

Eric N. Olson’s group has recently discovered [4] a conserved micropeptide, myoregulin, encoded by RNA that had been misannotated as noncoding. Myoregulin is a skeletal muscle-specific micropeptide (46 amino acids) that forms a single transmembrane alpha helix within the membrane of the sarcoplasmic reticulum (SR), where it finely regulates Ca^2+^ handling interacting with the SR Ca^2+^ ATPase (SERCA). Intriguingly, myoregulin displays a robust structural resemblance to phospholamban and sarcolipin, which inhibit SERCA activity in the heart and in slow-type and developing skeletal muscle [5]. Specific inhibition of myoregulin could have beneficial effects not only in terms of contractility but also in the control of systemic energy homeostasis [6]. The fact that putative long noncoding RNA may harbor hidden micropeptides had been suggested by recent genome-wide analyses [7]. However, heretofore the microproteome has largely been overlooked in gene annotations. Therefore, scanning the microproteome embedded in the annotated noncoding RNA will certainly be a major field of research in the next years. The hunt is open!

## 4. Diabetes Ask1ng for Trouble: Terminating microRNA Biogenesis in ER Stress

Highlight by Gaetano Santulli

The functional link between endoplasmic reticulum (ER) stress and miRNA has been demonstrated as a crucial step in the pathophysiology of diabetic embriopathy [8]. Peixin Yang’s group at the University of Maryland elegantly unveiled [8] that Ask1 (apoptosis signal-regulating kinase 1), an oxidative stress-responsive kinase activated in maternal diabetes, is a fundamental component of the unfolded protein response [9]. Indeed, Ask1 deletion reduced the endoribonuclease activity of IRE1α (inositol-requiring enzyme 1α), leading to reduced X-box binding protein 1 (XBP1) mRNA splicing, a key marker of ER stress [10]. Moreover, the Ask1-IRE1α signalosome triggers a rapid decay of several miRNAs, including miR-17, miR-34a, and miR-96, mechanistically involved in ER stress. These findings open new exciting research fields in the analysis of the complex interplay between miRNAs and diabetes mellitus [11].

## 5. Worms Silence Like a Plant

Highlight by R. Keith Slotkin.

Transposable element (TE) activity is repressed in eukaryotes by positive feedback loops of small RNA production and activity. Plant small interfering RNAs (siRNAs) are dependent on Dicer and RNA-dependent RNA Polymerase (RdRP) proteins, while in animals, Dicer- and RdRP-independent small RNAs, called piRNAs, repress TEs. Sarkies *et al*., recently demonstrated that in contrast to the well-studied *C. elegans*, most nematode species do not have piRNAs and rely on Dicer and RdRP amplified siRNAs to silence TEs [12]. Therefore, some animals have plant-like TE suppression, and the addition of the piRNA system allows for the evolutionary loss of TE-regulating Dicers and RdRPs.

## 6. Targeted RNA Sequencing Reveals the Extraordinary Depth and Complexity of the Transcriptome

Highlight by John S. Mattick

Transcriptional profiling is bedeviled by the domination of the data by highly expressed RNAs, which means that cell- or stage-specific transcripts are undersampled, especially in complex tissues like the brain. In a recent paper in *Nature Methods* [13], Clark *et al*., used a new technique called RNA CaptureSeq, which employs bead arrays to capture transcripts from specific genomic regions—like a molecular microscope—to identify large numbers of previously unknown long noncoding RNAs (lncRNAs), as well as many new isoforms of mRNAs. They also show that the methodology is quantitative and equal or superior to conventional RNAseq across most of the expression range.

## 7. Methylation Directs Microprocessor Activity

Highlight by Ulf Andersson Ørom

In miRNA biogenesis, how the specificity of the recognition by Microprocessor is mediated to avoid processing of non-miRNA hairpins is not fully understood. In their study, Alarcon *et al*., present evidence that m6A RNA methylation occurs on pri-miRNA transcripts, in proximity to the stem of the miRNA hairpin, and modulates the processing of pri-miRNAs [14]. The authors show that m6A on selected pri-miRNA transcripts is important for microprocessor mediated cleavage and miRNA biogenesis, and enhances the level of mature miRNAs. A model where DGCR8 recognition dependent on m6A of pri-miRNA transcripts occurs, putatively in collaboration with an unknown factor, is presented.

## 8. Dissecting RNA Skeleton *in Vivo*

Highlight By Jian-Hua Yang

The structures of RNA molecules, the skeletons bound by trans-acting RNA-Binding proteins (RBPs), are essential for their function and regulation. Two recent studies developed new methods to dissect RNA secondary structures *in vivo*. Sugimoto and colleagues developed hiCLIP (RNA hybrid and individual-nucleotide resolution ultraviolet crosslinking and immunoprecipitation) to probe RNA structures interacting with RBPs and revealed a high prevalence of intra-molecular and long-range RNA duplexes recognized by Staufen 1 (STAU1) RBP in human 293 cells [15]. In a separate study, Spitale and colleagues presented icSHAPE (*in vivo* click selective 2’-hydroxyl acylation and profiling experiment) to decode structural dynamics of RNAs in mouse embryonic stem cells and accurately predicted RBP binding sites and N^6^-methyladenosine (m^6^A) modification spots from structural footprints [16]. Future application of these two methods to various cells or tissues and RBPs will reveal a full characterization of RNA structurome and provide new insights into the post-transcriptional gene regulation.

## 9. Highlights of the Paper by Lauressergues *et al.* in *Nature*

### 9.1. Don’t Call Me Non-Coding

Highlight by Manuela Ferracin

MicroRNAs are generated from longer precursor transcripts called primary miRNAs (pri-miRNAs), either in plants and animals. Pri-miRNAs are thought to be non-coding intermediates in miRNA processing. These transcripts, indeed, are processed rapidly inside the nucleus to generate pre-miRNAs (in animals) or miRNA duplexes (in plants). But is this all? Lauressergues *et al*. reported in *Nature* recently that some pri-miRNAs contain short open reading frames (ORFs) that can potentially generate peptides [2]. For some of them, from two different plants, they synthesized the predicted peptides, that they called miPEPs, and detected their distribution using specific antibodies. The miPEPs discovered in the study were able to enhance their corresponding pri-miRNA transcription. So the question is: how many coding non-coding RNAs are we ignoring?

### 9.2. Hunting for Small in Eukaryotic Genomes

Highlight by Florent Hubé

A recent report by Lauressergues *et al*., points to regulatory peptides encoded by unpredictable short ORFs sheltered in pri-miRNAs [2]. In a feed-forward loop, they enhance expression of the pri-miRNA and hence the mature regulatory miRNAs, potentiating target genes silencing. This discovery comes when hidden levels of genomic information are unveiled gradually. Micropeptides, smaller than the initial size-limit for proteins, can play key roles in biological process. Likewise, the frontier between coding and non-coding is increasingly blurred since the discovery of bifunctional RNAs that have both abilities. Micropeptides from non-coding pre-miRNAs are like a double issue, yet they provide a new perspective in functional genomics.

### 9.3. Are Non-Coding RNAs Indeed Non-Coding?

Highlight by Zofia Szweykowska-Kulinska

A very exciting discovery was done by the group of Jean-Philippe Combier from the University of Toulouse and CNRS Castanet-Colosan in France [2]. They showed that primary transcripts of many plant microRNAs encode short regulatory peptides that increase microRNA levels by stimulating their transcription. Plant pri-miRNAs can be very long ranging from several hundred nt in length up to more than 3000 nt [17]. For a long time, an open question was why plant *MIR* genes take so long encoding just a small 21 nt long molecule? Now the riddle is partially solved: at least part of the non-coding pri-miRNAs turned to be bifunctional: producing miRNAs and coding small peptide mRNAs.

## 10. What Do You Get for 493 Billion RNA Fragments?

Highlight by Daniel Gautheret

A group led by Arul Chinnayan from the University of Michigan, Ann Harbor, answers this question in the March issue of *Nature Genetics* [18]. Their curation of over 7200 RNA-seq libraries from human tissues and cell lines, provided by The Cancer Genome Atlas (TCGA) and other large scale projects, expands the number of transcribed genes by at least 30% from current official annotations. The bulk of the new RNA-producing loci are lncRNA genes, whose total number now reaches 58,000 by their count. Using dedicated bioinformatics, the authors provide an interesting array of filters to prioritize lncRNAs, based on sequence conservation, density of disease-causing variants and differential expression.

## 11. AGO6 Is Involved in Endo-siRNA Mediated Silencing

Highlight by Franck Vazquez

Besides the genetically well-characterized RdDM pathway which implicates Pol IV, Pol V, RDR2 and 23–24-nt long siRNAs in *Arabidopis thaliana*, an independent endo-siRNA pathway is involved in the establishment of transcriptional gene silencing (TGS) of TAS (*trans*-acting siRNA) loci and certain transcriptionally active transposable elements (TEs). The group of Keith Slotkin just showed that this so-called RDR6-RdDM pathway depends on direct guiding of AGO6 to TE DNA targets by 21–22-nt [19]. This study contributes to better understand the RDR6-RdDM pathway and adds a new example of the complexity of plant small RNA pathways.
